# Evaluation of a Newly Developed Live Attenuated Vaccine Candidate Against *Lawsonia intracellularis*

**DOI:** 10.3390/vaccines14010015

**Published:** 2025-12-22

**Authors:** Huixing Lin, Xuan Liu, Jingzhi Yuan, Ning Xiao, Hong Zhou, Hongjie Fan

**Affiliations:** 1 MOE Joint International Research Laboratory of Animal Health and Food Safety, College of Veterinary Medicine, Nanjing Agricultural University, Nanjing 210095, China; linhuixing@njau.edu.cn (H.L.);; 2 College of Animal Science, Anhui Science and Technology University, Chuzhou 233100, China; 3 Jiangsu Co-Innovation Center for the Prevention and Control of Important Animal Infectious Diseases and Zoonoses, Yangzhou University, Yangzhou 225009, China

**Keywords:** porcine proliferative enteropathy, *Lawsonia intracellularis*, attenuated vaccine

## Abstract

**Background/Objectives**: *Lawsonia intracellularis* (*L. intracellularis*) is an important intestinal pathogen that causes porcine proliferative enteropathy (PPE) in swine production worldwide. Currently, only a few commercially available vaccines are available for PPE prevention. **Methods**: In this study, an attenuated *L. intracellularis* variant of JS-G90 was obtained through subculturing of *L. intracellularis* JS isolates in McCoy cells for 90 generations, and its immune response was evaluated in pigs. **Results**: The results demonstrated that pigs who underwent intragastric administration of JS-G90 had lower fecal bacterial shedding and no histopathological lesions, indicating that it was safe in pigs. Therefore, JS-G90 was selected to develop the attenuated PPE vaccine. The immune response of JS-G90 in pigs was further evaluated based on fecal bacterial shedding, histopathological lesions, and humoral and cell-mediated immune responses following challenge with pathogenic *L. intracellularis*. The results revealed that JS-G90 significantly decreased the copies of *L. intracellularis* in rectal swabs containing feces and ileum infection (*p* < 0.001), reduced histopathological lesions in the ileum, and elicited non-specific humoral (IgG and sIgA) and cell-mediated immune responses (*p* < 0.001) compared with the challenge control and mock groups. **Conclusions**: In conclusion, the attenuated vaccine JS-G90 is safe and induced humoral and cell-mediated immune responses in pigs against pathogenic *L. intracellularis* infection. It may serve as an effective strategy for preventing and controlling PPE.

## 1. Introduction

Porcine proliferative enteritis (PPE), caused by *Lawsonia intracellularis* (*L. intracellularis*), is an important intestinal disease in swine production worldwide [1]. The economic losses caused by PPE are mainly related to increased feeding and medication costs, reduced weight gain, and longer times to reach slaughter weight [2]. The prevention and control of PPE mainly rely on commercially available vaccines [3,4]. Antimicrobial therapy has become the preferred method to control the disease during PPE outbreaks. Previous studies have shown that high doses of antimicrobial drugs such as tiamulin, tylosin, chlortetracycline, lincomycin, and olaquindox have some effect against *L. intracellularis* [5]. However, antibiotics effective against *L. intracellularis* can only control PPE in the short-term due to intermittent shedding of the bacteria; furthermore, antibiotic usage in animals may result in antibiotic residues [5]. Moreover, it is difficult to screen effective antibiotics using in vitro susceptibility testing promptly to treat a PPE outbreak, as *L. intracellularis* cannot grow on a cell-free medium in vitro [6].

There are two main types of vaccines against *L. intracellularis* that have been marketed: attenuated vaccines and inactivated vaccines [4,7,8]. Both types provide effective immune protection to prevent PPE; however, pigs receiving the attenuated vaccine do not reduce fecal bacterial shedding after challenge, and inactivated vaccines do not induce mucosal immune responses [9,10]. Mucosal immunity may play a role in preventing the invasion and intracellular proliferation of enteric pathogens such as *L. intracellularis*. Therefore, it is generally considered that live attenuated vaccines administered locally are more effective in inducing mucosal immune responses than inactivated vaccines administered systemically [11]. Given the nature of *L. intracellularis* as a local intestinal infection, oral live attenuated vaccines are expected to provide better efficacy in resisting *L. intracellularis* infection. Consequently, this study aimed to obtain non-pathogenic *L. intracellularis* variants via multiple generations in cell culture and evaluate the susceptibility and virulence of pigs to *L. intracellularis* JS isolates after 30, 60, and 90 generations in vitro. Consequently, an attenuated vaccine candidate against *L. intracellularis* was developed and its immune response in pigs was evaluated. This study was motivated by previous studies that developed a live attenuated Lawsonia vaccine, with improved evaluation of cellular and humoral immune responses.

## 2. Materials and Methods

### 2.1. Bacterial Isolates, Cell Line, and Culture Conditions

*L. intracellularis* JS isolates were previously obtained from the intestines of a gilt that had acute bloody diarrhea and were preserved in our lab [12]. The isolates were passaged in continuous generations in McCoy cells, as described in [13]. At each generation, the bacteria were purified and preserved in DMEM with 10% fetal bovine serum (FBS) and 10% dimethyl sulfoxide (DMSO), then stored at −80 °C. The number of *L. intracellularis* in each inoculum was assessed via direct counting after indirect immunoperoxidase (IPX) staining of serial 10-fold dilutions prepared in sterile phosphate-buffered saline, as previously described, with some modifications [13]. Briefly, the bacteria infected during pure culture of the *L. intracellularis* were detected by preparing 10-fold serial dilutions of the pure culture in DMEM, coating 24-well glass slides with 10 μL of each dilution, drying at 37 °C, fixing with ice-cold 4% paraformaldehyde, permeabilizing with 0.3% TritonX-100, and staining with a specific monoclonal antibody against *L. intracellularis* Omp2 [14]. The numbers of *L. intracellularis* were counted under light microscopy. Finally, DMEM was used to dilute the specified concentration according to the counting results. A commercial live attenuated *L. intracellularis* vaccine was purchased from Aniheal company, Nanjing, China and used according to the manufacturer’s instructions.

### 2.2. Determination of Safety of L. intracellularis JS Isolates After In Vitro Generations

Eighty 6–7-week-old female Yorkshire pigs were confirmed to be negative for *L. intracellularis* using quantitative real-time PCR (qPCR), as previously described [15], and the indirect enzyme-linked immunosorbent assay kit (ELISA, Biostone, Southlake, TX, USA). All pigs were fed a base diet and housed in the animal experiment center, which provided clean conditions, including a circulating ventilation system and controlled temperature and humidity. They were randomly divided into four groups (*n* = 20 per group). These groups were intragastrically inoculated with 20 mL of purified JS isolates containing approximately 5 × 10^8^ *L. intracellularis* at generations 30 (JS-G30), 60 (JS-G60), and 90 (JS-G90), and PBS (negative control), respectively. The pigs were monitored daily for changes in clinical parameters (appetite, movement, mental state, and body temperature) up to 21 days post-inoculation (dpi). The pigs’ weight was individually recorded at the beginning and end of the experiment to determine the average daily weight gain (ADWG). Fecal samples were collected from all pigs on days 0, 7, 14, and 21 dpi. All pigs were euthanized at 21 dpi and evaluated for PPE lesions. Intestinal samples from the ileum were also collected. The non-pathogenic generation of *L. intracellularis* was used to prepare a vaccine based on the growth rate, fecal bacterial shedding, and microscopic lesions.

### 2.3. Evaluation of Immune Response of Newly Developed Attenuated Vaccine

Eighty 6–7-week-old female Yorkshire pigs were confirmed to be negative for *L. intracellularis* using quantitative real-time PCR (qPCR), as previously described [15], and the indirect enzyme-linked immunosorbent assay kit (ELISA, Biostone, Southlake, TX, USA). All pigs were fed a base diet and housed in the animal experiment center, which provided clean conditions, including a circulating ventilation system and controlled temperature and humidity. The pigs were randomly divided into four groups (*n* = 20 per group). Group 1 and group 2 pigs were intragastrically vaccinated with 20 mL of JS-G90 (2 × 10^8^ bacteria (JS-G90 group)) and the commercial live attenuated vaccine (following the manufacturer’s instructions (commercial vaccine group)), respectively. Group 3 (no-vaccine (challenge control group)) and group 4 (no-vaccine and no-challenge (mock group)) pigs were intragastrically treated with 20 mL of DMEM. Groups 1, 2, and 3 were challenged intragastrically with 20 mL of a pathogenic *L. intracellularis* JS isolate (generation 10) containing approximately 1 × 10^9^ *L. intracellularis* via gavage at 21 days post-vaccination. The pigs were monitored daily for body condition, diarrhea, and abnormal behavior. The pigs’ weight was individually recorded at the beginning and end of the experiment to determine the ADWG. Rectal swabs containing feces and blood samples were collected at 0, 7, 14, and 21 days post-challenge (dpc). On 21 dpc, all the pigs were necropsied and evaluated for typical PPE lesions. Intestinal samples from the ileum were collected.

### 2.4. Quantification of L. intracellularis Fecal Bacterial Shedding

Total DNA was extracted from rectal swabs containing feces (0.2 g) using a Fecal Genomic DNA Kit (Vazyme Biotech Co., Nanjing, China). The concentration and quality of the DNA were then determined with a Nanodrop ND-1000 spectrophotometer (Thermo Fisher Scientific, Waltham, MA, USA). Quantitative real-time PCR (qPCR) was performed to quantify the *L. intracellularis* using the following primers: 5′-GCTGTGGATTGGGAGAAATC-3′ (forward) and 5′-CAAGTTGACCAGCCTCTGC-3′ (reverse), as previously described [15]. Absolute quantification was calculated using a standard curve for *L. intracellularis* and expressed as copy numbers of the *aspA* gene of *L. intracellularis* per gram of the rectal swab containing feces.

### 2.5. Detection of L. intracellularis-Specific IgG Using Indirect ELISA

The serum antibody response was measured using indirect ELISA [16]. Briefly, 96-well plates were coated with 100 μg/mL of purified Omp2 protein of *L. intracellularis* in a volume of 100 µL per well and incubated overnight at 4 °C. Then, the wells were blocked with 5% skimmed milk in phosphate-buffered solution (PBS (pH 7.4)) for 2 h at 37 °C. The sera were diluted at a 2-fold serial dilution and added to the wells in triplicate for 1 h at 37 °C. The plates were incubated with HRP-conjugated goat anti-pig IgG at ratios of 1:5000 for 1 h at 37 °C. Following incubation, the color reaction was developed by adding 3,3′,5,5′-tetramethylbenzidine (TMB) for 15 min, and the reaction was stopped with 2 mol/L sulfuric acid. The optical density (OD) was measured using a microtiter plate reader (Tecan, Männedorf, Switzerland) at 450 nm. The cutoff value was determined when the OD_450nm_ ratio (OD_450nm_ of sample/OD_450nm_ of negative serum) was above 2.1.

### 2.6. Quantification of Secretory IgA in Ileal Mucosa

The concentration of the total secretory IgA (sIgA) in the ileal mucosa was measured. The ileum samples were opened longitudinally at 21 dpc, and the mucosa was scraped using a glass slide. The mucosa samples were then added to PBS containing 0.2 mol/L ethylenediaminetetraacetic acid (EDTA), and the supernatants were collected following centrifugation at 10,000 rpm for 10 min at 4 °C. The sIgA was measured using the pig sIgA ELISA Kit (Elabscience, Wuhan, China), according to the manufacturer’s instructions.

### 2.7. Histopathological and Immunofluorescence Assay for L. intracellularis

After the pigs were sacrificed at 21 dpc, the intestinal epithelial tissue slides were fixed in 10% neutral buffered formalin for at least 24 h. Subsequently, the formalin-fixed tissues were cut into 4 µm sections. Histopathological assay was conducted following the protocols, as previously described [17]. Meanwhile, paraffin-embedded blocks were used for immunofluorescence assay (IFA) with an *L*. *intracellularis*-specific Omp2 monoclonal antibody 4D9 [14] to examine *L. intracellularis* colonization of the ileum, according to previously reported methods [7]. The morphology of the intestinal villi, epithelial cell proliferation, and *L. intracellularis* colonization in the ileum were examined using H&E staining and IFA. The pathologist was blinded to the sample identity to evaluate the tissue sections.

### 2.8. Measurement of Cell-Mediated Immune Responses

The concentrations of total interferon-gamma (IFN-γ) and total interleukin-12 (IL-12) in the serum samples were assessed using ELISA Kits (Xuanyabio, Shenzhen, China) at 0, 7, 14, and 21 dpc. Moreover, the lymphocyte transformation assay of ileum lymphocytes in each group was determined using the MTT [3-(4,5-dimethylthiazol-2-yl)-2,5-diphenyltetrazolium bromide] method. Briefly, the ileum of each pig was collected at 21 dpc, and lymphocytes were separated using an intestinal mucosal tissue lymphocyte separation solution (Biolab, Beijing, China). Then, the lymphocytes were suspended in RPMI 1640 medium containing 10% FBS, 50 U/mL of penicillin, and 50 g/mL of streptomycin. The lymphocyte suspensions were added to 96-well plates at a concentration of 5 × 10^5^ cells per well. Subsequently, the T-lymphocyte mitogen phytohemagglutinin M (PHA-M) was added at a final concentration of 100 μg/mL. The cell cultures were incubated at 37 °C in 5% CO_2_ for 72 h, during which the mitogen produced its maximal effect on DNA synthesis. The cells were then pulsed with MTT and incubated at 37 °C in 5% CO_2_ for 4 h. The results were presented as a stimulation index (SI), calculated as follows: SI = (OD_570nm_ stimulated well − OD_570nm_ blank well)/(OD_570nm_ unstimulated well − OD_570nm_ blank well).

### 2.9. Statistical Analysis

The results were analyzed and graphed using GraphPad Prism 9. The data are expressed as the mean ± SD (standard deviation) of at least 3 independent experiments. Statistical differences were assessed using Student’s *t*-test and one-way analysis of variance (ANOVA) with Tukey’s test. A value of *p* < 0.05 was considered significant.

## 3. Results

### 3.1. Determination of Safety of Different Generations of L. intracellularis JS Isolates

**Figure 1:**
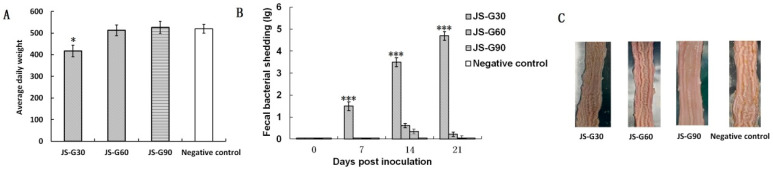
Screening of the non-pathogenic *L. intracellularis* variant of JS isolates. (**A**) Average bodyweight of pigs after inoculation with generations 30, 60, and 90 of JS isolates. (**B**) Quantification of *L. intracellularis* in rectal swabs containing feces. Real-time quantitative PCR (qPCR) was conducted to determine the amount of *L. intracellularis* DNA in fecal samples collected at 0, 7, 14, and 21 days post-inoculation. The results were reported as threshold cycle numbers (Ct) and converted to copies per gram. Assays were concluded after 40 cycles. The data represent the mean ± SD of 3 independent experiments. (**C**) The pigs were necropsied at 21 dpi for evaluation of pathological changes in the ileum. *, *p* < 0.05; and ***, *p* < 0.001 (compared with the mock group).

The clinical parameters of the pigs were observed daily post-intragastrical administration of different generations of *L. intracellularis*. The results indicated that pigs in all groups (*n* = 20) showed normal appetite, movement, and mental states throughout this study. Pigs in the JS-G30 group had a transient increase in body temperature that lasted for 1–2 days, while pigs in the JS-G60, JS-G90, and negative control groups maintained normal body temperature consistently. The pigs were weighed at the beginning and end of the experiment to assess the effects on ADWG of different generations of *L. intracellularis* JS isolates. No significant difference in the ADWG of the JS-G60 and JS-G90 groups compared with the negative control group was observed, while the ADWG of the JS-G30 group was significantly decreased compared with the other three groups (*p* < 0.05; Figure 1A). Particularly, pigs in the JS-G60 and JS-G90 groups showed significantly lower fecal bacterial shedding at 7, 14, and 21 dpi (*p* < 0.001; Figure 1B) compared with the JS-G30 group. Furthermore, no microscopic lesions of the ileum were observed in the JS-G60 and JS-G90 groups (Figure 1C), and pigs in the negative control group did not shed a detectable level of *L. intracellularis* in rectal swabs containing feces throughout this study. These results show that JS-G60 and JS-G90 are safe after intragastric inoculation. To ensure safety, JS-G90 was further tested as a live attenuated vaccine against *L. intracellularis*.

### 3.2. Fecal Bacterial Shedding and Histopathology Detection Post-Challenge

The pigs’ growth performance, fecal bacterial shedding, histopathological lesions, and humoral and cell-mediated immune responses were assessed to evaluate the immune response associated with JS-G90 following challenge with pathogenic *L. intracellularis*.

**Figure 2:**
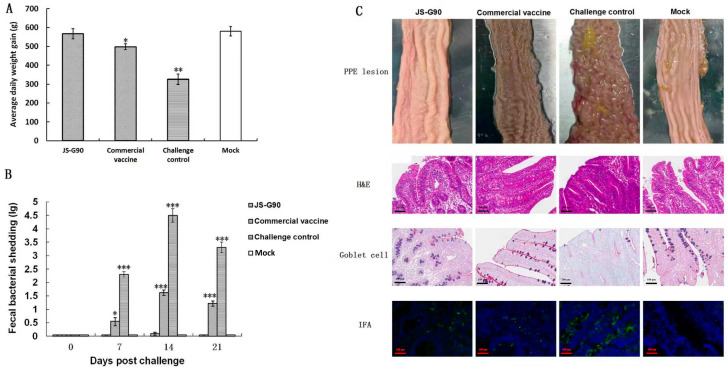
Growth performance, levels of *L. intracellularis* fecal bacterial shedding, histopathology evaluation, and immunofluorescence assay after challenge. (**A**) Effect of JS-G90 on the growth rate of pigs. (**B**) Estimated *L. intracellularis* per gram in rectal swabs containing feces at 0, 7, 14, and 21 days post-challenge (dpc). The data represent the mean ± SD of 3 independent experiments. (**C**) Histopathological evaluation. Pigs were necropsied at 21 dpc, and H&E staining was performed for histopathologic assessment. No obvious gross lesions or microscopic lesions of ileitis were observed in the groups vaccinated with the commercial vaccine and JS-G90 compared with the control group. The IFA results showed that *L. intracellularis* bacteria were significantly reduced in the JS-G90 group and commercial vaccine group compared with the challenge control group. *, *p* < 0.05; **, *p* < 0.01; and ***, *p* < 0.001 (compared with the mock group).

The average daily weight gain in the JS-G90 group (*n* = 20) was significantly higher (*p* < 0.01) from 0 dpc to 21 dpc compared with the challenge control group (*n* = 20), and was significantly higher (*p* < 0.05) than that of pigs in the commercial vaccine group (Figure 2A). The qPCR results showed that the peak levels of fecal bacterial shedding were observed at 14 dpc. However, fecal bacterial shedding in the JS-G90 group and the commercial vaccine group was significantly lower than in the challenge control group (*p* < 0.001). Moreover, the bacterial shedding in the JS-G90 group was lower than that in the commercial vaccine group at 14 and 21 dpc (*p* < 0.001; Figure 2B). The morphology of the intestinal villi, epithelial cell proliferation, and *L. intracellularis* colonization in the ileum were examined using H&E staining and IFA. Pigs in the JS-G90, commercial vaccine, and mock groups showed complete intestinal villi and crypt structures at 21 dpc. However, lesions in the challenge control group were more evident in the ileum, characterized by altered morphology and severe proliferation of intestinal crypt lining cells (enterocytes) that formed multiple cell layers (Figure 2C).

### 3.3. Humoral Immune Response Analysis Post-Challenge

**Figure 3:**
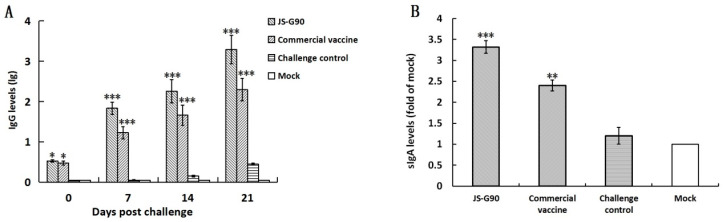
Levels of *L. intracellularis*-specific antibodies after challenge. (**A**) The levels of IgG in the serum of different groups after challenge. Sera collected at 0, 7, 14, and 21 dpc were evaluated with indirect ELISA. The data represent the mean ± SD of 3 independent experiments. (**B**) Pigs were sacrificed at 21 dpc, and the ilea were collected to detect the levels of sIgA according to the instructions of a commercially available ELISA kit. The data represent the mean ± SD of 3 independent experiments. *, *p* < 0.05; **, *p* < 0.01; and ***, *p* < 0.001 (compared with the mock group).

According to the ELISA procedure, the IgG titers in serum collected at 0, 7, 14, and 21 dpc were investigated. As shown in Figure 3A, the pigs immunized with JS-G90 induced IgG antibody responses at 0 dpc (21 days after immunization). The post-challenge antibody levels of pigs in the JS-G90 and commercial vaccine groups were significantly higher (*p* < 0.001) than in the challenge control group and the mock group at 7, 14, and 21 dpc. However, no significant difference was found between the groups vaccinated with JS-G90 and the commercial vaccine.

Mucosal immunity plays a critical role in preventing enteric pathogens’ invasion and intracellular proliferation, such as *L. intracellularis* [11]. The total sIgA was detected in the ileum content collected at 21 dpc to explore the effect of JS-G90 immunization on the mucosal immune response. As shown in Figure 3B, the levels of total sIgA in the groups vaccinated with JS-G90 (*p* < 0.001) and the commercial vaccine (*p* < 0.01) were significantly higher than in the challenge control group and the mock group. Moreover, the total sIgA in the JS-G90 group was significantly higher than that in the commercial vaccine group (*p* < 0.05).

### 3.4. Cell-Mediated Immune Response Analysis Post-Immunization

**Figure 4:**
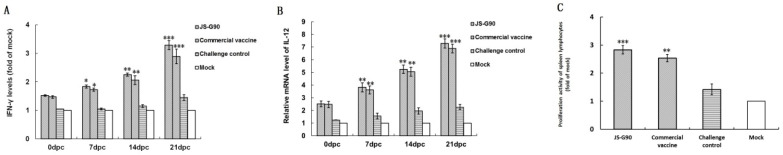
Detection of cytokine responses after challenge. (**A**) IFN-γ in sera collected at 0, 7, 14, and 21 dpc. The data represent the mean ± SD of 3 independent experiments. (**B**) IL-12 in sera collected at 0, 7, 14, and 21 dpc. The data represent the mean ± SD of 3 independent experiments. (**C**) The proliferation activity of ileum lymphocytes in pigs was determined using MTT at 21 dpc. The data represent the mean ± SD of 3 independent experiments. *, *p* < 0.05; **, *p* < 0.01; and ***, *p* < 0.001 (compared with the mock group).

The concentrations of total IFN-γ in serum at 0, 7, 14, and 21 dpc were monitored using a commercial ELISA kit to evaluate the cell-mediated immune response. As shown in Figure 4A, the levels of total IFN-γ in the JS-G90 and commercial vaccine groups significantly increased at 7 (*p* < 0.05), 14 (*p* < 0.01), and 21 (*p* < 0.001) dpc compared with the challenge control group and the mock group.

As shown in Figure 4B, the JS-G90 and commercial vaccine groups had increased mRNA levels of total IL-12 related to the cell-mediated immune response compared with the challenge control group and the mock group at 7 (*p* < 0.01), 14 (*p* < 0.01), and 21 (*p* < 0.001) dpc. Furthermore, total IL-12 expression did not significantly differ between the groups vaccinated with JS-G90 and the commercial vaccine.

T-lymphocyte activation after stimulation with PHA-M was measured by analyzing ileum lymphocytes isolated from each group at 21 dpc. The T-lymphocyte proliferative activities in the JS-G90 group (*p* < 0.01) and commercial vaccine group (*p* < 0.05) were significantly higher than in the challenge control group and the mock group (Figure 4C). The results suggested that JS-G90 induced a non-specific *L. intracellularis* cell-mediated immune response in the pigs.

## 4. Discussion

A previous study reported attenuation of the virulence of *L. intracellularis* between 20 and 40 cell generations in vitro [18]. This study repeated previous studies that developed a live attenuated *Lawsonia* vaccine, but with improved evaluation of cellular and humoral immune responses. In this study, a non-pathogenic *L. intracellularis* variant of JS-G90 isolates was obtained through subculturing in McCoy cells, in particular, after generation 90. The results showed that JS-G90 did not affect the growth performance of the pigs; particularly, pigs inoculated with JS-G90 had less fecal bacterial shedding, less bacterial colonization of the ileum, and no histopathological lesions. Therefore, JS-G90 was used to develop a candidate attenuated vaccine against *L. intracellularis*. As expected, pigs vaccinated with JS-G90 and the commercial vaccine did not show crypt epithelial hyperplasia after the bacterial challenge. Although fecal bacterial shedding in the JS-G90 and commercial vaccine groups was significantly lower, the bacterial shedding in the JS-G90 group was significantly lower than that in the commercial vaccine group at 7, 14, and 21 dpc. However, abundant *L. intracellularis* DNA and antigens were found in the cytoplasm of hyperplastic epithelial cells. These results indicate that JS-G90 is safe as it caused a reduction in the shedding of *L. intracellularis*—to a certain extent—after the challenge, and effectively mitigated *L. intracellularis* infection in pigs.

Specific IgG may play an important role in resisting *L. intracellularis* infection [7]. In this study, the antibody levels in serum were further detected to evaluate the efficacy of JS-G90. The serum IgG level, not antigen-specific, in the vaccinated JS-G90 group was significantly higher than in the control group post-challenge. However, lower IgG was detected in the control group at 14 and 21 days post-challenge, consistent with a previous study [19]. These data suggest that JS-G90-vaccinated pigs induced a non-specific *L. intracellularis* humoral response post-challenge.

According to Go et al. [20], IFN-γ may play a role in preventing PPE lesion development in *L. intracellularis*-infected pigs. As an obligate intracellular bacterium, replication of *L. intracellularis* in the intestinal epithelium may induce IFN-γ- and cell-mediated immune responses against the pathogens, similar to other intracellular pathogens [21]. This study showed that the IFN-γ response in serum was significantly increased in the commercial vaccine and JS-G90 groups compared with the control group, indicating that JS-G90 elicited a cell-mediated immune response. This study thus supports the findings of the abovementioned studies [20,22].

The relative mRNA expression related to cell-mediated immune response in all groups was further examined. IL-12 is a heterodimeric cytokine produced by antigen-presenting cells to regulate the activation and differentiation of lymphocytes [23]. Previous studies have shown that IL-12 appears to be a prerequisite for optimal Th1 responses, plays a crucial role in promoting cell-mediated immunity against intracellular pathogens, and induces proliferation and differentiation of naive CD4+ T cells into Th1 cells [24,25]. In this study, the mRNA expression of the immune-modulatory cytokine IL-12 was significantly increased in the experimental groups immunized with the commercial vaccine and JS-G90 compared with the control group. These results reveal that innate and adaptive immune responses were generated in JS-G90-vaccinated pigs.

## 5. Conclusions

In summary, a newly developed attenuated candidate vaccine against *L. intracellularis* named JS-G90 was successfully developed via multiple generations (90). JS-G90 induced humoral and cell-mediated immune responses in pigs. This study provides valuable information for developing a candidate commercial vaccine against PPE.

## Data Availability

All data used during this study are available from the corresponding author upon request.

## References

[1] McOrist S., Barcellos D., Wilson R.J.P.J. (2003). Global patterns of porcine proliferative enteropathy. Pig J..

[2] Paradis M.-A., Gebhart C.J., Toole D., Vessie G., Winkelman N.L., Bauer S.A., Wilson J.B., McClure C.A. (2012). Subclinical ileitis: Diagnostic and performance parameters in a multi-dose mucosal homogenate challenge model. J. Swine Heal. Prod..

[3] McOrist S., Smits R.J. (2007). Field evaluation of an oral attenuated *Lawsonia intracellularis* vaccine for porcine proliferative enteropathy (ileitis). Vet. Rec..

[4] Kroll J.J., Roof M.B., McOrist S. (2004). Evaluation of protective immunity in pigs following oral administration of an avirulent live vaccine of *Lawsonia intracellularis*. Am. J. Vet. Res..

[5] Pereira C.E.R., Resende T.P., Vasquez E., Marshall-Lund L., Guedes R.M.C., Gebhart C.J. (2019). In vitro antimicrobial activity against equine *Lawsonia intracellularis* strains. Equine Vet. J..

[6] Wattanaphansak S., Pereira C.E.R., Kaenson W., Assavacheep P., Tantilertcharoen R., Resende T.P., Barrera-Zarate J.A., de Oliveira-Lee J.S.V., Klein U., Gebhart C.J. (2019). Isolation and in vitro antimicrobial susceptibility of porcine *Lawsonia intracellularis* from Brazil and Thailand. BMC Microbiol..

[7] Roerink F., Morgan C.L., Knetter S.M., Passat M.H., Archibald A.L., Ait-Ali T., Strait E.L. (2018). A novel inactivated vaccine against *Lawsonia intracellularis* induces rapid induction of humoral immunity, reduction of bacterial shedding and provides robust gut barrier function. Vaccine.

[8] Jacobs A.A.C., Harks F., Pauwels R., Cao Q., Holtslag H., Pel S., Segers R. (2020). Efficacy of a novel intradermal *Lawsonia intracellularis* vaccine in pigs against experimental infection and under field conditions. Porc. Health Manag..

[9] Jacobs A.A.C., Harks F., Hazenberg L., Hoeijmakers M.J.H., Nell T., Pel S., Segers R. (2019). Efficacy of a novel inactivated *Lawsonia intracellularis* vaccine in pigs against experimental infection and under field conditions. Vaccine.

[10] Riber U., Heegaard P.M., Cordes H., Ståhl M., Jensen T.K., Jungersen G. (2015). Vaccination of pigs with attenuated *Lawsonia intracellularis* induced acute phase protein responses and primed cell-mediated immunity without reduction in bacterial shedding after challenge. Vaccine.

[11] Guedes R.M., Gebhart C.J. (2010). Evidence of cell-mediated immune response and specific local mucosal immunoglobulin (Ig) A production against *Lawsonia intracellularis* in experimentally infected swine. Can. J. Vet. Res..

[12] Xiao N., Li J., Li M., Zhou H., Lin H., Fan H. (2022). Isolation and In Vitro cultivation of *Lawsonia intracellularis* from China. Vet. Microbiol..

[13] Vannucci F.A., Wattanaphansak S., Gebhart C.J. (2012). An alternative method for cultivation of *Lawsonia intracellularis*. J. Clin. Microbiol..

[14] Xiao N., Li J., Li M., Hu Y., Lin H., Fan H. (2021). Preparation and Characterization of a New Monoclonal Antibody Specific Against *Lawsonia intracellularis* and Its Application in Indirect Immunofluorescence and Immunocytochemistry Assay. Front. Vet. Sci..

[15] Wattanaphansak S., Gebhart C.J., Anderson J.M., Singer R.S. (2010). Development of a polymerase chain reaction assay for quantification of *Lawsonia intracellularis*. J. Vet. Diagn. Investig..

[16] Zhang C., Zhou H., Lin H., Fan H. (2024). Development and Application of Indirect ELISA Kits for Antibody Detection of Porcine Proliferative Enteropathy. Sci. Agric. Sin..

[17] Guedes R.M., Gebhart C.J., Winkelman N.L., Mackie-Nuss R.A., Marsteller T.A., Deen J. (2002). Comparison of different methods for diagnosis of porcine proliferative enteropathy. Can. J. Vet. Res..

[18] Vannucci F.A., Beckler D., Pusterla N., Mapes S.M., Gebhart C.J. (2013). Attenuation of virulence of *Lawsonia intracellularis* after in vitro passages and its effects on the experimental reproduction of porcine proliferative enteropathy. Vet. Microbiol..

[19] Helm E.T., Burrough E.R., Leite F.L., Gabler N.K. (2021). *Lawsonia intracellularis* infected enterocytes lack sucrase-isomaltase which contributes to reduced pig digestive capacity. Vet. Res..

[20] Go Y.Y., Lee J.K., Ye J.Y., Lee J.B., Park S.Y., Song C.S., Kim S.K., Choi I.S. (2005). Experimental reproduction of proliferative enteropathy and the role of IFN-gamma in protective immunity against *Lawsonia intracellularis* in mice. J. Vet. Sci..

[21] Monack D.M., Bouley D.M., Falkow S. (2004). Salmonella typhimurium persists within macrophages in the mesenteric lymph nodes of chronically infected Nramp1+/+ mice and can be reactivated by IFNgamma neutralization. J. Exp. Med..

[22] Smith D.G., Mitchell S.C., Nash T., Rhind S. (2000). Gamma interferon influences intestinal epithelial hyperplasia caused by *Lawsonia intracellularis* infection in mice. Infect. Immun..

[23] Glassman C.R., Mathiharan Y.K., Jude K.M., Su L., Panova O., Lupardus P.J., Spangler J.B., Ely L.K., Thomas C., Skiniotis G. (2021). Structural basis for IL-12 and IL-23 receptor sharing reveals a gateway for shaping actions on T versus NK cells. Cell.

[24] Müller U., Köhler G., Mossmann H., Schaub G.A., Alber G., Di Santo J.P., Brombacher F., Hölscher C. (2001). IL-12-independent IFN-gamma production by T cells in experimental Chagas’ disease is mediated by IL-18. J. Immunol..

[25] Hamza T., Barnett J.B., Li B. (2010). Interleukin 12 a key immunoregulatory cytokine in infection applications. Int. J. Mol. Sci..

